# What is needed for nurses to work with evidence-based practice? A qualitative study

**DOI:** 10.1080/10376178.2024.2369660

**Published:** 2024-07-01

**Authors:** Jeltje Giesen, Annick Bakker-Jacobs, Anneke van Vught, Marjolein Berings, Hester Vermeulen, Getty Huisman-de Waal

**Affiliations:** a Radboud University Medical Centre, Radboud Institute for Health Sciences, IQ Healthcare, Nijmegen, The Netherlands; b HAN University of Applied Sciences, School of Allied Health, Department of Nutrition and Health, Nijmegen, The Netherlands; c Radboud University Medical Centre, Radboudumc Health Academy, Nijmegen, The Netherlands; d Radboud University Medical Centre, Surgical Department, Nijmegen, The Netherlands

**Keywords:** Community health services, evidence-based nursing, healthcare, hospitals, leadership, nurses, students

## Abstract

**Background:**

Transformation of healthcare is necessary to ensure patients receive high-quality care. Working with the evidence-based practice (EBP) principles enables nurses to make this shift. Although working according to these principles is becoming more common, nurses base their actions too much on traditions and intuition. Therefore, to promote EBP in nursing practice and improve related education, more insight into nurses’ needs is necessary to overcome existing EBP barriers.

**Objective:**

To identify the current needs to work with EBP principles among hospital and community care nurses and student nurses.

**Design:**

A qualitative, exploratory approach with focus group discussions

**Methods:**

Data was collected between February and December 2020 through 5 focus group discussions with 25 nurses and student nurses from a hospital, a community care organisation, and nursing education schools (bachelor and vocational). Data were analysed using reflexive thematic analysis, and the main themes were synchronised to the seven domains from the Tailored Implementation for Chronic Diseases (TICD) checklist.

**Results:**

Nurses and student nurses experience EBP as complex and require more EBP knowledge and reliable, ready-to-use evidence. They wanted to be facilitated in access to evidence, the opportunity to share insights with colleagues and more time to work on EBP. The fulfilment of these needs serves to enhance motivation to engage with evidence-based practice (EBP), facilitate personal development, and empower nurses and student nurses to take more leadership in working according to EBP principles and improve healthcare delivery.

**Conclusion:**

Nurses experience difficulties applying EBP principles and need support with their implementation. Nurses’ and student nurses’ needs include obtaining more EBP knowledge and access to tailored and ready-to-use information. They also indicated the need for role models, autonomy, incentives, dedicated time, and incorporation of EBP in daily work practice.

## Impact statement

An understanding of the needs of nurses and student nurses regarding the application of EBP principles will help to empower them and to enable institutions to be more responsive to their needs. This, in turn, will result in the promotion of evidence-based quality of care.

## Plain language summary

Healthcare must change to maintain high quality care for patients. Following the principles of Evidence-Based Practice (EBP) can contribute to this transformation. However, nurses often find it difficult to implement EBP insights in their practice because they face challenges such as lack of time, skills, knowledge and resources. Therefore, it is necessary to explore their needs for working according to EBP principles to help overcome the barriers they currently experience. Through group discussions with nurses and student nurses, it has been identified that they have specific needs in relation to the application of EBP. Nurses and student nurses experience EBP as complex and therefore need more knowledge and training in EBP. They would like more time to work on EBP in daily practice and the opportunity to discuss it with colleagues. In addition, they would like more tailored ready-to-use evidence that is easily accessible and, if possible, in their native language. Meeting these wishes helps to increase their motivation to work with EBP and contributes to their personal growth. They therefore require organisational support in terms of time to implement EBP, training and access to all available knowledge. Which is crucial for increasing the use of EBP and helping them to take leadership in applying EBP in practice. Doing so will allow them to provide better care.

## 1. Introduction

Transforming healthcare is necessary to ensure patients receive high-quality care (Robertson-Preidler et al., 2017). This need is led by the increasing demand for care, the aging population, continuous labour shortage, and professionals leaving healthcare prematurely (Visser et al., 2021; WHO, 2015). Nurses constitute the largest group of care providers and administer the majority of care (WHO, 2020). Therefore, they can play a pivotal role in the transformation of care. Nurses can improve the quality of care by assessing and changing how they provide care (Visser et al., 2021). This can be achieved by de-implementing time-consuming and potentially harmful low-value care and incorporating evidence-based practice (EBP) principles into their daily nursing routines (Burston et al., 2014; Verkerk et al., 2018). EBP is a worldwide recognised approach that integrates the best available evidence, clinical expertise, and patient values and preferences when making care decisions. Integrating EBP helps to sustain and improve nursing care by strengthening nurses’ care delivery foundation and enabling them to reflect systematically on their practice(Baker, 2017; Melnyk et al., 2016).

Over the years, EBP has become more common in nursing practice, and its principles have been incorporated into the undergraduate nursing curriculum (Lam & Schubert, 2019). Present-day student nurses can identify fundamental EBP competencies, such as searching for evidence, but struggle with disseminating best practices or integrating EBP changes (Lam & Schubert, 2019). In addition, it is known that student nurses find it hard to apply their EBP knowledge in practice after graduation and experience a lack of support from nurses in utilising and applying EBP principles (Lam & Schubert, 2019; Skela-Savič et al., 2020). This is not surprising as nurses themselves asses their EBP skills as below average and often encounter difficulties integrating EBP into their daily routine, resulting in experiencing resistance to working with EBP principles (Kerr & Rainey, 2021; Lehane et al., 2019; Melnyk et al., 2016). In addition, nurses base their practice too much on traditions and intuition and struggle with incorporating patient preferences in daily practice (van Belle et al., 2018; Zwakhalen et al., 2018). Two systematic reviews confirmed and identified further barriers nurses encounter while using EBP. These barriers relate to their skills, knowledge, resources, professional relationships, and the absence of authority (Jabonete & Roxas, 2022; Kajermo et al., 2010). The combination of high workloads and insufficient time during work hours are important factors hindering nurses’ engagement in EBP (Jabonete & Roxas, 2022; Maaskant et al., 2013; Mallion & Brooke, 2016).

To promote quality of care and utilisation of EBP, it is necessary to explore other aspects nurses and student nurses experience beyond the limitations of time. Insights can contribute to filling gaps in the nursing education curriculum and to support the development of integrated teaching strategies to promote EBP knowledge and skills of student nurses and nurses in daily practice (Horntvedt et al., 2018). Therefore, the aim of this study was to identify the perceived needs of nurses and student nurses for improving engagement in the use of EBP in hospital and community care settings.

## 2. Methods

### 2.1. Design

The study used a qualitative, exploratory approach with focus group discussions to gain in-depth information and insights into the needs of hospital and community care nurses and student nurses to work with EBP principles. Conducting focus group discussions enabled the researchers to discover what groups of nurses felt and thought about this specific topic and supported them in sharing and discussing it with each other (Holloway & Galvin, 2016). The standards for reporting qualitative research (SRQR) were used to report the study.

### 2.2. Study setting and recruitment

Nurses from hospitals or community care organisations offering services to patients in urban and rural settings and student nurses from bachelor’s and vocational nursing schools in the eastern region of the Netherlands were purposefully selected to participate in focus group discussions. In the Netherlands, vocational nursing education is more practical and focuses on the delivery of care in daily practice based on EBP knowledge. In the bachelor’s nursing education, nurses are trained to manage the care process and to use EBP to improve healthcare delivery.

The principal investigator (JG) approached nurses using a personal email, while student nurses were approached during lectures, and it was made clear that participating was voluntary. Five homogeneous groups with 4–10 participants were created to ensure that a proper and effective discussion could be facilitated and that participants felt comfortable speaking out. In total, there were 25 participants (21 female, four male), aged 18–48 years, and with work experience of 6 months to 12 years (see Table 1). Initially, the focus group discussions were planned on site. However, only digital meetings were allowed for a period due to the government's proclaimed COVID-19 restrictions. Conducting focus group discussions has advantages as it often gives a lower participation threshold. However, a disadvantage is that interaction is often less natural in an online environment (Flayelle et al., 2022). Despite this, the researchers did not get the impression that it affected the study outcomes.

**Table 1.** Characteristics of participants.

Participant	Setting	Gender	Age	Work experience (years)
**Focus group 1:** Parttime nursing students following an undergraduate education. Already with a vocational degree and work experience				
01	Hospital	Male	31	11
02	Community care	Female	32	12
03	Hospital	Female	27	4
04	Hospital (Psychiatry)	Female	22	2
05	Community care	Female	34	*
06	Hospital (Psychiatry)	Male	48	*
07	*	Female	34	8
08	Hospital	Female	23	3
09	Community care	Female	27	*
**Focus group 2:** Full-time nursing students following an undergraduate education				
10	NA	Female	22	NA
11	NA	Female	20	NA
12	NA	Female	20	NA
13	NA	Female	18	NA
14	NA	Female	*	NA
**Focus group 3:** Hospital Nurses				
15	Hospital	Female	37	15
16	Hospital	Female	47	27
17	Hospital	Male	27	6
18	Hospital	Female	36	9
**Focus group 4:** Community care nurses				
19	Community care	Female	27	7
20	Community care	Female	25	0.5
21	Community care	Female	31	4
22	Community care	Female	28	6
**Focus group 5:** Full-time nursing students following a vocational education				
23	NA	Female	19	NA
24	NA	Male	20	NA
25	NA	Female	21	NA

*Not provided; NA: not applicable

### 2.3. Data collection

All focus group discussions were conducted by the main researcher (JG) between February and December 2020 and observed by a researcher (MvdH) or educational science student (MM). These researchers were registered nurses and health scientists (MSc) with qualitative research experience. They had no connection to the included participants. Before the focus group discussions commenced, a guide was developed. This guide was discussed with an advisory panel of four nursing researchers, six nurses from hospital and community care settings, three experts in nursing education, and three advisors from professional nursing associations. The focus group discussions were semi-structured to allow the researchers to anticipate specific discussion topics. At the initiation of each discussion, a moment was taken to facilitate introductions, ensure that all participants understood the purpose of the discussions, and get to know each other. They all started with the question: *‘Could you tell us where you search for evidence for working EBP principles?*’ and focused on where nurses and student nurses search for evidence, their challenges, and their needs for EBP use in daily practice (see table 2). The focus group discussions lasted an hour on average and was audio-recorded. They were verbally transcribed by a specialised company, and a member check was conducted with a summary of the transcript, to which all nurses agreed. Furthermore, the observing researchers took field notes that included information about participants’ body language, tone of voice, and other details that stood out, which were used in the analysis.

**Table 2:** Focus group discussion guide.

Question	Related topics
*1. Could you tell us about where you search for evidence for working with the EBP principles?*	Research evidence - Scientific journals - Nursing journals - Social media - Nursing Networks Clinical expertise Patient preferences
*2. What are the challenges you face for working with the principles of EBP?*	Known EBP-Barriers 1 Insufficient time in work to implement new ideas. 2 No time during work to read research articles. 3 Not having sufficient authority to change procedures change in patient care. 4 Statistical analyses are not presented intelligibly. 5 The relevant literature is not available in one place. 6 Facilities are inadequate. 7 Other staff do not support implementation. 8 Physicians do not want to cooperate in implementation. 9 Nurses are not aware of research. 10 Not feeling capable of assessing the quality of research
*3. What do you need to improve working with the EBP principles in daily practice?*	Research evidence - Searching for research evidence - Availability of evidence - Presentation of evidence Clinical expertise - Sharing clinical expertise - Knowledge Patient preferences

### 2.4. Data analysis

The data collection and data analysis of the focus group discussions was an iterative process. For analysis, we used a reflexive thematic analysis that was conducted moving forward through the following six phases: 1. Familiarisation with the data; 2. generating initial codes; 3. generating themes; 4. reviewing potential themes; 5. defining and naming themes; and 6. producing the report (Braun & Clarke, 2023). ‘All the transcripts independently open coded by the main researcher (JG) or research assistants (ABJ and AO) and ATLAS.Ti version 8.4.20 was used. Codes were collaborative and reflexive obtained to achieve richer interpretations of meaning. When necessary, a third researcher was consulted. As coding progressed, a correlation was found with the seven domains of the factors outlined in the Tailored Implementation for Chronic Diseases (TICD) checklist (Flottorp et al., 2013). The TICD checklist is comprehensive, generic, and the authors suggested that it could be used for a broader patient category. We decided to synchronise the theme names to increase the transferability of the results and increase usability by researchers and others involved in quality improvement projects (Flottorp et al., 2013).

### 2.5. Ethical considerations

The research ethics committee of the Radboudumc determined that ethical approval was not required under Dutch law (CMO no. 2020-6136). Nevertheless, all participants signed an informed consent stating they were informed about the purpose of the study, voluntary participation, and the right to withdraw from the study at any given time without disclosing a reason, and that confidentiality and anonymity of recordings and transcripts were assured.

## 3. Results

**Figure 1:**
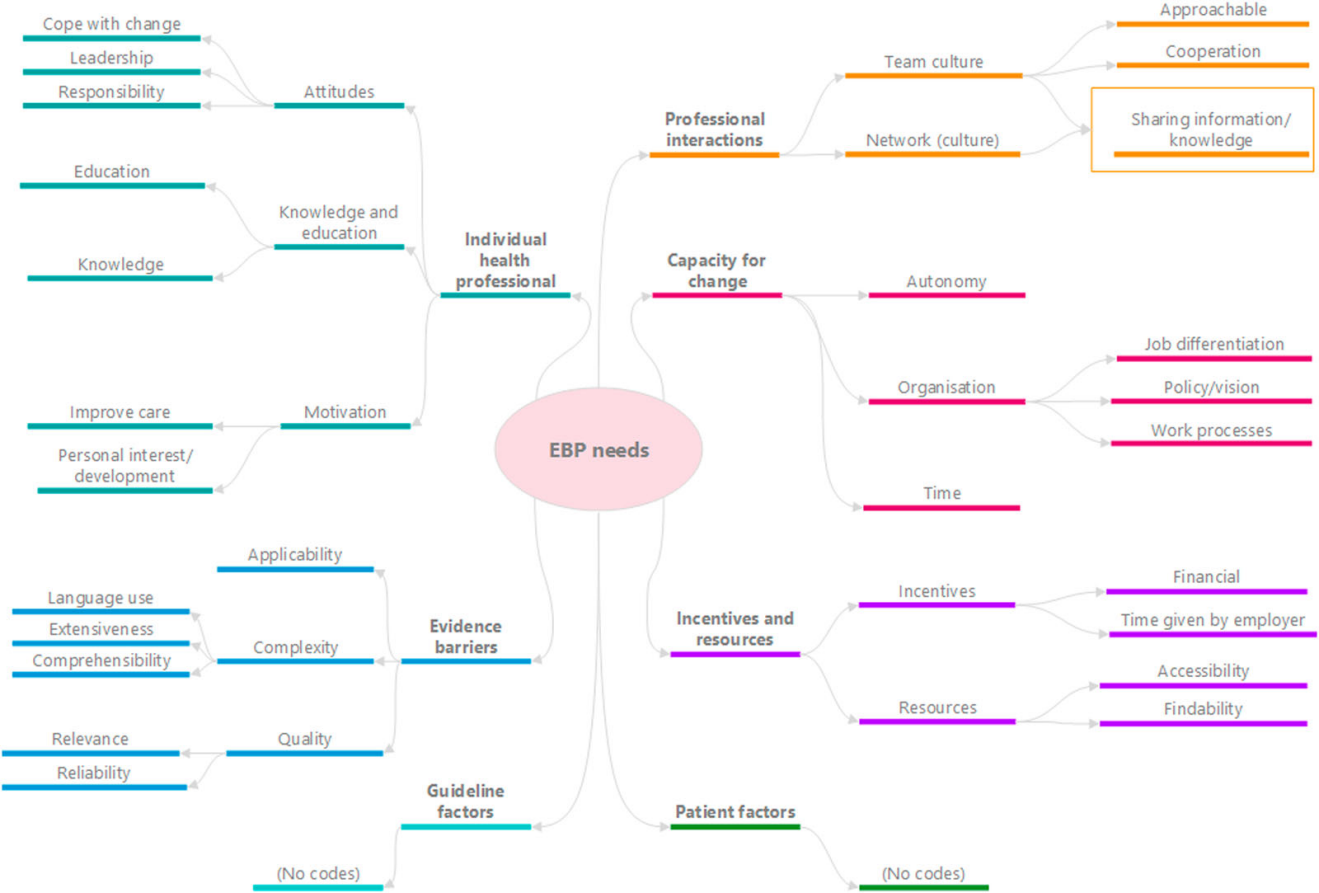
Mindmap EBP needs.

All codes from the analyses could be categorised under five domains of the TICD checklist, which will be explained in this results section. No codes could be linked to the domains ‘guideline factors’ and ‘patient factors’ (see Figure 1). To enhance clarity, the codes are formatted in bold to highlight the discussed concepts.

### 3.1. Evidence barriers

In this domain, three distinct themes emerged: complexity, applicability, and quality of the evidence. Participating nurses and student nurses expressed the desire to develop their knowledge to work with scientific articles and EBP principles. They said it is essential to utilise these skills to prevent diminishment over time. Participating nurses experience evidence in a non-native language as complex. Their understanding of evidence could be enhanced with summaries in their native language. Participating student nurses said they encountered fewer difficulties with the English language of research, as they frequently use it daily and get more comprehensive EBP education.

Although preferable, all research translated into Dutch feels a bit excessive since I watch everything in English on Netflix. [Student nurse]

All participants experienced research findings as too scientific and were overwhelmed by the quantity of available evidence, resulting in doubts about their EBP capabilities and skills to interpret statistical results. Therefore, they expressed the need for readily applicable and tailored evidence about the field of nursing work, which enhances usability, reduces reading time, and helps overcome the feeling of evidence extensiveness.

It can be a lot because research outcomes can be contradicting, which can make me clueless. There is a need for evidence that works well, has been assessed in practice, and is immediately applicable. [Community care nurses]

All participants agreed that the tailored evidence should be reliable, as they all find it important to use high-quality information. To assess the quality, the tailored evidence should give details about the information’s source, research methodologies, authors, publication year, and sponsors.

I always look at how research is conducted. Do they use a small number of participants, or is it of low quality? Then it is less trustworthy. [Student nurse]

### 3.2. Individual health professionals

This domain includes three themes: motivation, attitude, and knowledge to work with the EBP principles. All participants told to have a positive attitude toward EBP and were motivated to improve care. They agreed that working with EBP is part of their job and essential for delivering the best possible care. Nevertheless, participating nurses and student nurses acknowledged that research enthusiasm is personal and understandably not universally shared among colleagues. Participating student nurses especially encounter problems with this and need colleagues with a positive attitude toward EBP collaboration.

Colleagues must be open to EBP. At internships, I often hear, ‘We always do it like this.’ They are not open to changes. [Student nurse]

To increase their EBP motivation, participating nurses agreed to require role modelling from colleagues or the opportunity to pursue further education or refresher courses, which is also beneficial for their personal development. This education should focus on how and where to find evidence and get experience with implementing EBP principles.

Education is the first step to getting questions answered, and knowledge about an EBP model can help get started independently. [Hospital nurse]

Both participating nurses and bachelor student nurses were inclined to collaborate as they perceive it as the responsibility of all nurses to enhance care quality through EBP. They agreed about the necessity for increased leadership and the consideration of individual roles. Due to their experiences with hands-on colleagues, assigning EBP to a bachelor-educated nurse or a document manager. This was confirmed by expressions of vocational student nurses that told to consider EBP more as a bachelor nurse task.

### 3.3. Professional interactions

This domain captures two themes: team culture and network (culture). Participating nurses and student nurses both experienced it as a team culture to first ask colleagues or workgroups for solutions. The need to obtain a quick answer takes precedence, and they rely on their colleague's knowledge.

I ask colleagues, and they often have an answer. I don't know whether that is EBP, but I rely on their expertise. [Hospital nurse]

Most participating nurses wanted the opportunity to share knowledge within organisational networks or beyond, which is already facilitated in some hospitals. However, they experienced colleagues’ reluctance to embrace change, and the fear of receiving constructive criticism is an obstacle to this knowledge exchange. Additionally, student nurses emphasise the necessity for support and cooperation to work with EBP as their suggestions for care changes are not always appreciated, and they feel they are disregarded.

### 3.4. Incentives and resources

This domain focused on incentives from the organisation and resources to work with the principles of EBP. More time to work on EBP is an incentive addressed by all participating nurses. They indicate that organisations focus too much on providing bedside care, especially in the community care setting.

Due to staff shortages, we do not have time to work on EBP. Organisations focus on maximising bedside care and hours that can be claimed. [Community care nurse]

Participating nurses wanted time or financial compensation when searching for evidence. Participating community care nurses expressed the need for more resources as finding and accessing evidence is often challenging for them. Go-getters are creative in obtaining reliable evidence. However, others turn to non-scientific Internet pages like Facebook, which offers quick and readable information.

### 3.5. Capacity for change

This domain contains three themes: organisation, lack of time, and autonomy. Both nurses and student nurses expressed confusion regarding expectations regarding EBP caused by an unsuccessful implementation of job differentiation. They said that EBP is mentioned in their job descriptions but not incorporated into daily work processes.

EBP is not part of my daily work routines. It's not that I think, oh, let me work on EBP. [Community care nurse]

Participating nurses found it difficult to incorporate EBP due to a high workload, staff shortages, and insufficient time. They expressed the need for more time and autonomy to put EBP into practice and that discussing and solving care problems positively influences team spirit and quality of care. The use of EBP can be supported by a guiding person, a well-defined EBP vision, and an organisational policy.

## 4. Discussion

The objective of this study was to explore the needs of nurses and student nurses with regard to the application of EBP principles, which are important to facilitate the transformation that is needed in healthcare. The analysis yielded insights into the desire for nurses and student nurses to take ownership and to show leadership in working with EBP principles. To foster a culture of learning, it is essential that nurses and student nurses are supported by their organisations through provision of time, resources for locating evidence, and clear expectations and acknowledgement of applying EBP in practice. There is need for tailored and high-quality evidence related to nursing care, that is easily accessible. Furthermore, nurses require education to refine their EBP skills and attitudes, which can be used to provide more appropriate care.

The findings indicate that increased ownership and being able to take leadership are essential for fostering positive attitudes toward utilising EBP principles and it is therefore recommended that resources should be allocated towards this endeavour. These insights are supported by a cross-sectional survey study involving 356 hospital nurses (Stokke et al., 2014). This study found that having a positive attitude, sufficient knowledge, and being involved in working groups increases the implementation of EBP. Participation of nurses in EBP can be improved by the promotion of self-efficacy, outcome expectancy, active nurse leadership and supportive work environments, as was found in another cross-sectional survey study involving 439 hospital nurses (Hoegen et al., 2022). In addition, a qualitative focus group study with 26 nurses from different settings confirms that nurses need the opportunity to control of their nursing practice, can work autonomously, and they have to be clinically competent in order to use EBP and improve patient care (Kieft et al., 2014). Furthermore, an ethnographic study with 12 semi-structured interviews shows that nursing leaders knowledgeable about EBP methodology and research experience can help to promote EBP in organisations (Bahlman-van Ooijen et al., 2023).

Besides the opportunity to show more leadership by providing autonomy, it is important that healthcare organisations provide nurses with access to knowledge, in order to meet the EBP principles of using the best available evidence (Baker, 2017). In addition, nurses need to be facilitated in time and be provided with a clear and unclouded vision on EBP from their organisation. This is consistent with an integrative review that includes 28 studies and a project evaluation with eight interviews, which underpins the important role of nurse managers as gatekeepers for the promotion of EBP in daily practice and for facilitating access to knowledge and resources (Bianchi et al., 2018; Friesen-Storms et al., 2017). Furthermore, it is essential to consider that an unsupportive work environment impedes nurses’ personal growth and their ability to reach their full EBP potential, as stated in the WHO guide on facilitating EBP in nursing (Jylhä et al., 2017). Consequently, it is imperative that organisations clarify their expectations regarding the utilisation of EBP in daily practice and continue job differentiation. Nevertheless, a multiphase qualitative interview study with 50 project managers in charge of job differentiation revealed that Dutch hospitals and community care organisations fail to differentiate sufficiently between vocational-educated nurses and bachelor-educated nurses (Van Kraaij et al., 2022). Therefore, it is important to put more emphasis on job differentiation to promote the use of EBP by nurses and student nurses.

Providing more education to nurses and student nurses contributes to job differentiation and will improve their EBP competencies. This is important, as applying EBP is challenging for nurses due to insufficient education or not using previously learned EBP skills. These educational needs are consistent with an intervention study that included 61 nurses and integrated two EBP education programs (Vaajoki et al., 2023). This study states that nurses require practical education and support to effectively recognise clinical issues and apply EBP in their daily patient care routines. Facilitating workplace learning for nurses can promote EBP in quality improvement in daily practice and encourage the utilisation of their clinical expertise (a pilar of EBP), as was found in action research in nursing homes that included twelve nursing teams (Lovink et al., 2022). Furthermore, it is recommended that educators pay attention to incorporating patients’ preferences, an EBP pillar that nurses did not mention in this research. Therefore, no code could be attached to the ‘patient factors’ of the TICD-checklist. Nevertheless, a qualitative study that performed 18 interviews with patients from the hospital in a community care setting about the needs of patients when creating a learning culture confirmed the importance of integrating patient preferences to establish a resilient nurse-patient relationship and to facilitate shared decision-making (Giesen et al.).

Finally, the study results reinforce the requirement for (further) bridging the gap between research, education, and nursing practice. It is important to adequately prepare student nurses during their studies for the utilisation of EBP in daily practice. A scoping review of 60 articles revealed that new nurses experience workplace incivility as they attempt to integrate into the profession and seek acceptance by the team (Hakvoort et al., 2022). This could result in adaptation of team traditions that do not contribute to quality of care. Furthermore, it is imperative to facilitate accessibility of evidence to enhance its applicability in practice. A systematic review of 28 articles provides evidence showing that nursing scientists should act as knowledge brokers, as they are able to establish and maintain partnerships, facilitate application of knowledge, generate new evidence, and promote EBP in nursing teams (Thompson & Schwartz Barcott, 2019). Although no specific codes could be linked to guideline factors, the need for more ready-to-use evidence can explain the suboptimal adherence to guidelines recommended by healthcare professionals, as was found in a systematic review of 235 studies (Grimshaw et al., 2006). Therefore, it is recommended that nurses and student nurses be provided with implementation strategies like the TICD checklist. This checklist is the result of a systematic review of 12 articles that focused on improving the practice of health care professionals. (Flottorp et al., 2013)

### 4.1. Strengths and limitations of the work

The strength of this study lies in providing an overview of nurses’ and student nurses’ needs regarding EBP. This is crucial for integrating the EBP principles into daily practice and supporting the cultivation of an EBP-learning culture within nursing teams. By involving student nurses, the aspirations of future nurses regarding EBP are captured.

The limitations of this study were governmental COVID-19 regulations that limited the number of participants to be included and forced a transition from in-person to digital focus group discussions. Furthermore, only hospital and community care nurses were included, neglecting perspectives from other healthcare settings. Incorporating FCD with managers or coaches could have offered a more comprehensive viewpoint, given that individuals might not always be fully aware of the underlying professional, team, and organisational culture. Lastly, it is possible that our results exhibited bias due to participants’ generally youthful age, and they were more inclined to participate due to their positive EBP attitude.

### 4.2. Recommendations for further research

Future research should focus on extending and validating our findings’ applicability by involving nursing managers and other nursing areas such as nursing homes, care for disabled people, and mental health facilities. In addition, research should focus on developing practical interventions that meet the need to work with EBP principles for nurses and organisations. Finally, research should ensure that findings are accessible, understandable, and usable to put into practice.

## 5. Conclusion

EBP has attracted more attention in nursing practice, and its importance is widely acknowledged. However, nurses experience difficulties applying EBP principles and need support with their implementation. Nurses’ and student nurses’ needs revolve around obtaining more EBP knowledge and access to tailored and ready-to-use information. Furthermore, they want the opportunity, time, and space to discuss issues and research findings with colleagues. Therefore, to enhance working with EBP principles within organisations, implementing strategies fostering and facilitating an EBP-learning culture is necessary, in which nurses are empowered to address EBP requirements and be encouraged to utilise and share their EBP knowledge in everyday practice. This will help to overcome EBP barriers and improve EBP decision-making, contributing to the delivery of high-quality care.

## CRediT authorship contribution statement

Jeltje Giesen: Conceptualisation, Methodology, Investigation, Formal analysis, Writing – original draft, Writing – review & editing. Annick Bakker-Jacobs: Conceptualisation, Methodology, Formal analysis, Writing – review & editing. Anneke van Vught: Funding acquisition, Conceptualisation, Methodology, Writing – review & editing, Supervision. Marjolein Berings: Funding acquisition, Conceptualisation, Methodology, Writing – review & editing, Supervision. Hester Vermeulen: Funding acquisition, Conceptualisation, Methodology, Writing – review & editing, Supervision. Getty Huisman-de Waal: Funding acquisition, Conceptualisation, Methodology, Formal analysis, Writing – review & editing, Project administration, Supervision.

## Data Availability

The analysed dataset from the study is available upon a reasonable request by the corresponding author.
